# Elucidating the Mechanisms of Influenza Virus Recognition by Ncr1

**DOI:** 10.1371/journal.pone.0036837

**Published:** 2012-05-15

**Authors:** Ariella Glasner, Antonija Zurunic, Tal Meningher, Tihana Lenac Rovis, Pinchas Tsukerman, Yotam Bar-On, Rachel Yamin, Adrienne F. A. Meyers, Michal Mandeboim, Stipan Jonjic, Ofer Mandelboim

**Affiliations:** 1 The Lautenberg Center for General and Tumor Immunology, Institute for Medical Research Israel Canada (IMRIC). Hebrew University-Hadassah Medical School, Jerusalem, Israel; 2 Department of Histology and Embryology/Center for Proteomics, Faculty of Medicine, University of Rijeka, Rijeka, Croatia; 3 Faculty of Life Sciences, Bar-Ilan University, Ramat Gan, Israel; 4 National Laboratory for HIV Viral Immunology National HIV and Retrovirology Laboratories, Public Health Agency of Canada, Winnipeg, Manitoba, Canada; 5 Central Virology Laboratory, Ministry of Health, Chaim Sheba Medical Center, Ramat-Gan, Israel; German Primate Center, Germany

## Abstract

Natural killer (NK) cells are innate cytotoxic lymphocytes that specialize in the defense against viral infection and oncogenic transformation. Their action is tightly regulated by signals derived from inhibitory and activating receptors; the later include proteins such as the Natural Cytotoxicity Receptors (NCRs: NKp46, NKp44 and NKp30). Among the NCRs, NKp46 is the only receptor that has a mouse orthologue named Ncr1. NKp46/Ncr1 is also a unique marker expressed on NK and on Lymphoid tissue inducer (LTI) cells and it was implicated in the control of various viral infections, cancer and diabetes. We have previously shown that human NKp46 recognizes viral hemagglutinin (HA) in a sialic acid-dependent manner and that the O-glycosylation is essential for the NKp46 binding to viral HA. Here we studied the molecular interactions between Ncr1 and influenza viruses. We show that Ncr1 recognizes influenza virus in a sialic acid dependent manner and that N-glycosylation is important for this binding. Surprisingly we demonstrate that none of the predicted N-glycosilated residues of Ncr1 are essential for its binding to influenza virus and we thus conclude that other, yet unidentified N-glycosilated residues are responsible for its recognition. We have demonstrated that N glycosylation play little role in the recognition of mouse tumor cell lines and also showed the *in-vivo* importance of Ncr1 in the control of influenza virus infection by infecting C57BL/6 and BALB/c mice knockout for Ncr1 with influenza.

## Introduction

Natural killer (NK) cells are innate cytotoxic lymphocytes that specialize in the defense against viral infection and oncogenic transformation [1], [2], [3]. Their activity is tightly regulated by cytokines and by signals derived from inhibitory and activating receptors [1], [2], [3]. Mostly MHC-I molecules, but also other proteins inhibit the activity of NK cells by binding to multiple NK inhibitory receptors that belong to the Ig super family and to the C-type lectin family [1], [2], [3]. The NK cell cytotoxicity is also positively controlled by activating receptors that include proteins such as CD16, NKp80, 2B4, NKG2D and the NCRs; NKp46, NKp44, and NKp30 [1], [2], [3]. Among the NCRs, NKp46 is the only receptor that has a mouse orthologe named Ncr1 [3], [4]. NKp46/Ncr1 is also a unique marker expressed on NK and on Lymphoid tissue inducer (LTI) cells [3]. It was implicated in the control of various viral infections [4], [5], [6], cancers [7], [8], [9], bacterial infection such as *Fusobacterium nucleatum* [10] and type I diabetes [11], [12].

Although the NCRs were discovered more than a decade ago [3], the identity of some of their ligands, particularly those recognized by NKp46/Ncr1 (such as the self, beta cell-derived [11], [12], bacterial [10]and tumor ligands [7], [8], [13]), is still obscure. Around 10 years ago we have identified viral hemagglutinin (HA) as a ligand for NKp46 and NKp44 [6], [9]. We have demonstrated that sialic acid residues play a critical role in the binding of both NKp44 and NKp46 to viral HA and showed that O-glycosylation of the sialic acid carrying residue threonine (located in position 225 of NKp46) is critical for the recognition of HA and of some tumor ligands _ENREF_1 [5]. Recently we showed that NKp46/Ncr1 interacts with a self-ligand expressed by beta cells in a sialic acid independent manner and that the Asn 216 and Thr 125 residues are important for beta cell recognition [12]. We further showed that glycosylation is not required for the NKp46/Ncr1 recognition of *Fusobacterium nucleatum* [10]. It is unknown how Ncr1 recognizes tumors and influenza viruses.

The importance of the interaction of Ncr1 with influenza virus was also confirmed *in-vivo* [4]. We have generated mice deficient for Ncr1 (*Ncr1gfp/gfp)* by replacing parts of the *Ncr1* gene with *GFP* and demonstrated that these mice succumb to influenza virus infection [4]. The critical role of Ncr1 in the recognition of influenza viruses was further emphasized by the recent discoveries demonstrating that influenza viruses developed various mechanisms to evade the Ncr1-mediated recognition. These include mutating the viral HA [14], infecting NK cells [15] and enhancing the inhibitory signals mediated by MHC class I [16], [17]. Furthermore, it was recently shown that both NKp46 and NKp30 bind to poxviral HA [18] and surprisingly, while the NKp46 recognition of poxviral HA leads to increased NK cell cytotoxicity, the binding of NKp30 leads to inhibition of NK cell killing via an unknown mechanism [18].

It is important to study the interactions between Ncr1 and HA and to compare it with the NKp46 interactions with HA in order to understand how previous and future work using mice models of influenza virus infection would be relevant in humans. Thus, we investigated here the mechanisms by which Ncr1 recognizes the influenza virus. Additionally, we demonstrate an Ncr1-mediated, dose dependent control of the PR8 influenza virus infection by infecting two inbred mouse strains, C57BL/6 and BALB/c knocked out for Ncr1 with influenza.

## Materials and Methods

### Mice, Killing Assays and *in-vivo* PR8 Infection

All animal work has been conducted according to relevant national and international guidelines. In accordance with the recommendations of the Weatherall report. The work was approved by the Hebrew University Medical School Ethic committee (Ethics number MD-10-12595-5). All the experiments were performed in a specific pathogen free unit of the Hebrew University Medical School (Ein-Kerem, Jerusalem) in accordance with the guidelines of the ethics committee. All experiments were performed using 6–8 weeks old female mice of the C57BL/6 or BALB/c backgrounds. The generation of the *Ncr1* knockout mice *Ncr1gfp/gfp* (KO) was described previously [4]. Both C57BL/6 and BALB/c mouse lines used were congenic lines generated from the original KO line (129/Sv background). For the killing and redirected killing assays, peripheral blood lymphocytes (PBLs) were harvested 18 hours following 200 µg poly(I):poly(C) in 200 µl PBS administered by i.p. injection to *Ncr1+/+* (WT) and *Ncr1gfp/gfp* (KO) mice of the C57BL/6 and BALB/c strains. The PBLs were incubated with EL4 cells and specific killing was determined as previously described [9]. Re-directed experiments were performed as previously described [9], briefly: P815 cells expressing Fc-γ receptor were coated with anti Ncr1 antibody to induce the re-directed NK cell killing for one hour on ice (to allow the binding of the anti-Ncr1 antibody to the P815 cells via its Fc) and NK cells were then added for additional 5 hours at 370C. For the *in-vivo* PR8 infection, mice were lightly anesthetized with 2% Isoflurane and intranasally inoculated with 4, or 40 hemagglutination units (HU) in 40 µl PBS. Mice were monitored thereafter daily for mortality. The human PR8 virus (A/PR/8/34) was used in all *in-vivo* and *in-vitro* experiments.

### Cells and *in-vitro* Coating with PR8 Influenza Virus

The cell lines used in PR8 influenza coating experiments were: The murine carcinogen induced lymphoma EL4 (ATCC), the virus-induced murine thymic lymphoma PD1.6 [14] and the human EBV transformed B cell lymphoma 721.221 (ATCC). The Canine kidney epithelial cells MDCK were used for influenza infection (ATTC). Propagation of the human influenza virus A/Puerto Rico/8/34 H1N1 (PR8) was performed as previously described [17]. Coating of the various cells with the virus was performed by incubating 1*106 cells in 2 ml complete medium with 10 µl or 20 µl (1000 HU over night at 37°C, 5% CO_2_. Infection of MDCK cells was performed with 1000 HU of the PR8 virus. Other cells used in this study were the murine B16 melanoma cells (ATCC), the murine Lewis Lung carcinoma cells D122 (ATCC), and the Mouse lymphoblast-like mastocytoma cell line P815 [17].

### Plasmids and Transfection

The putative glycosylation sites of Ncr1 were predicted by Biassoni et al [19]. For the generation of the WT and mutated Ncr1 fusion proteins, the sequence encoding the extracellular part of Ncr1 was amplified by PCR using the 5′ primer TAATAT GAATTC ATG CTG CCA ACA CTC ACT GCC (including *EcoRI* ) and the 3′ PRIMER TAATAT AGATC T TG GGT TGT GTG ATC CCA GA (including *BGLII*). These PCR-generated fragments were cloned into an expression vector containing a mutated Fc portion of human IgG1 (Fc mut pIRESpuro).

### Fusion Proteins, De-glycosylation, Abs, and flow Cytometry

The NKp46-D1-Ig and Ncr1-Ig fusion proteins were generated either in COS-7, or in HEK293T cells as previously described [9], [20]. Treatment of fusion proteins with neuraminidase (NA) was performed as described [5]. For removing N-glycosylation residues, 5 µg of NKp46-D1-Ig and Ncr1-Ig fusion proteins were incubated overnight with 3 µg Protein N-glycosidase F (PNGas F), in 50 µl PBS at 37°C. To remove O-glycosylation, 5 µg of NKp46-D1-Ig and Ncr1-Ig fusion proteins were incubated for 2 hours in 50 µl PBS at 37°C with a cocktail of enzymes: 3 µl α2 3,6,8,9-neuraminidase, 3 µl β1, 4-galactosidase, 3 µl endo-α-N-acetylgalactosaminidase, and 3 µl β1–2,3,4,6-N acetylglucosamnidase (Sigma Aldrich Israel). The staining of all cell lines by fusion proteins was visualized using secondary PE or APC conjugated goat anti-human mAbs. The anti-HA1 mAb H17-L2 is a kind gift from Jonathan W. Yewdell (NIH). The anti-Ncr1 mAb was generated by immunization of the Ncr1 KO mice with Ncr1-Ig.

### Generation of Mutations in Ncr1-Ig

The single point mutations in the Ncr1 proteins N139A, N216A N238A the double and the triple mutations were generated by using PCR-based, site-directed mutagenesis approach as previously described [5]. All products were cloned in frame with human IgG1 and the production of the fusion proteins was performed as previously described [20]. All fusion proteins were routinely tested for degradation on SDS PAGE gels, and no degradation products were detected.

### Statistical Analysis

Cytotoxicity at the different Effector to Target (E:T) ratios were assessed using repeated measures ANOVA, and survival was assessed using the Kaplan-Maier model and the Tarone-Ware test. P<0.05 was considered significant for all comparisons.

## Results

### The Ncr1 Glycosylation is Predicted to be Different from that of NKp46

**Figure 1:**
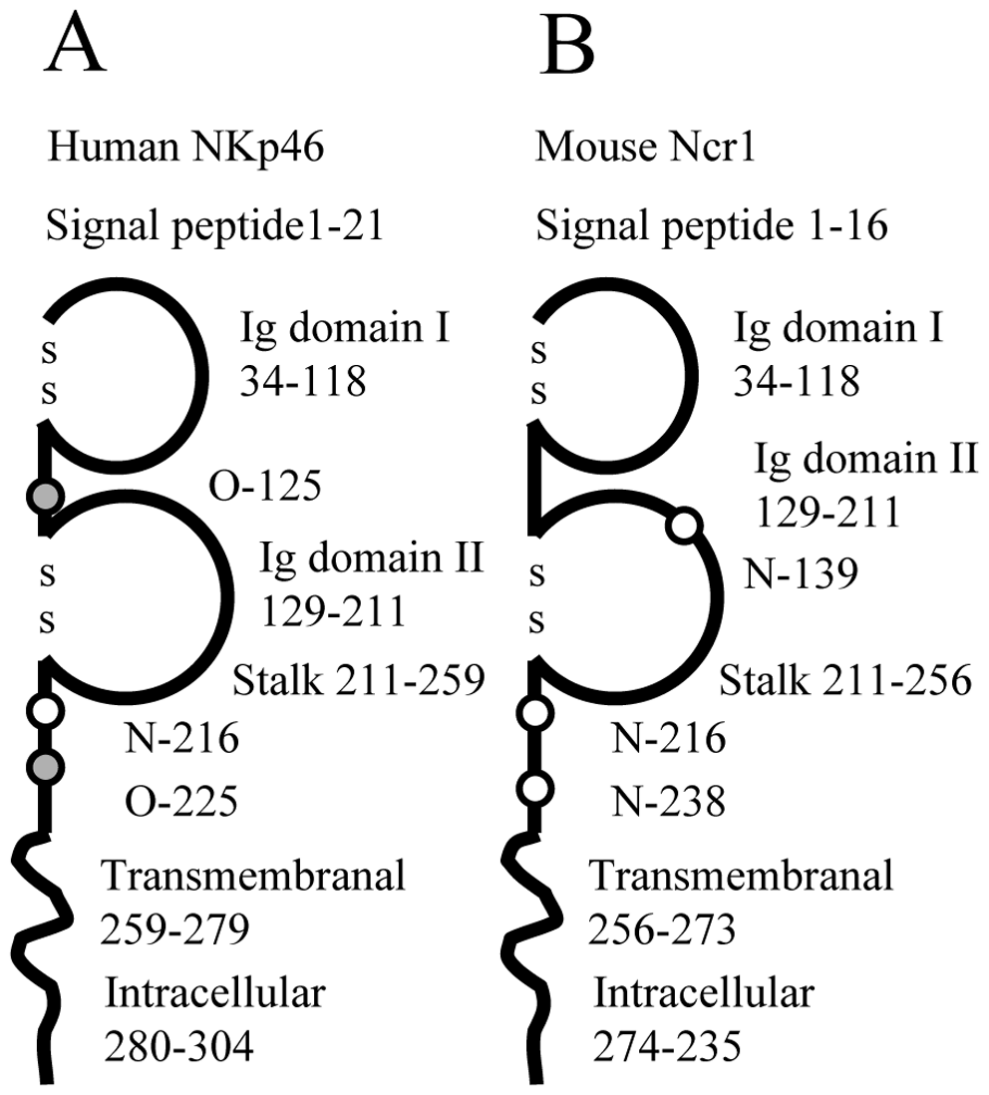
Human NKp46 and mouse Ncr1 structure. The figure shows a schematic description of the human NKp46 (A) and mouse Ncr1 (B) domains and glycosylation positions. White circle: N-linked glycosylation; Grey circle: O-linked glycosylation.

The initial aim of the current research was to study whether the mouse Ncr1 recognition of influenza virus is similar to that of the human NKp46. We have previously demonstrated that human NKp46 recognizes viral HA in a sialic acid dependent manner and that the recognition of HA by NKp46 is dependent on O-glycosylation of the Threonine residue located in position 225 of NKp46 [4], [5], [9]. Thus, we wondered whether the mouse orthologue of NKp46, Ncr1 would demonstrate a similar glycosylation-dependency. For that purpose we examined the structures of both Ncr1 and NKp46. NKp46 is a member of the Ig super family, consisting of a signal peptide region (residues 1–21), two Ig like domains (I; 34–118, II; 129–211), which are held by disulfide bonds (49↔98, 144↔190), a stalk region (211–259), a transmembranal domain (259–279) and an intracellular domain (280–304), (Fig. 1A). It is predicted to include three glycosylated residues, two O-glycosylations; on Thr 125 (this residue is located between the two Ig domains of NKp46) and on Thr 225 (located in the stalk region of NKp46) and one N-glycosylation residue, Asn 216, that is found in the stalk region of NKp46 (Fig. 1A). Ncr1 is also a member of the Ig super family. It consists of a signal peptide region, (position 1–16), two Ig like domains (I; 34–118, II; 129–211), which are held by disulfide bonds (49↔98, 144↔190), a stalk region (211–256), a transmembranal domain (256–273) and an intracellular domain (274–235), (Fig. 1B).

NKp46 and Ncr1 are substantially different with regard to their predicted glycosylation patterns as Ncr1 is not predicted to have O-glycosylated residues and is instead predicted to have three N-glycosilated residues ([19] and Fig. 1B). These include: Asn 139 in the membrane proximal Ig domain and two Asn residues located in positions 216 and 238 in the stalk region ([19], Fig. 1B).

Because we demonstrated that the O-glycosylated residue of NKp46 is critical for its HA recognition [4], [5], [9], the absence of O glycosylation in Ncr1 suggests that the Ncr1 recognition of the viral HA is probably different from that of NKp46.

### The Ncr1-Ig Binding to PR8-coated Cells is Sialic Acid, N-glycosylation-dependent

**Figure 2:**
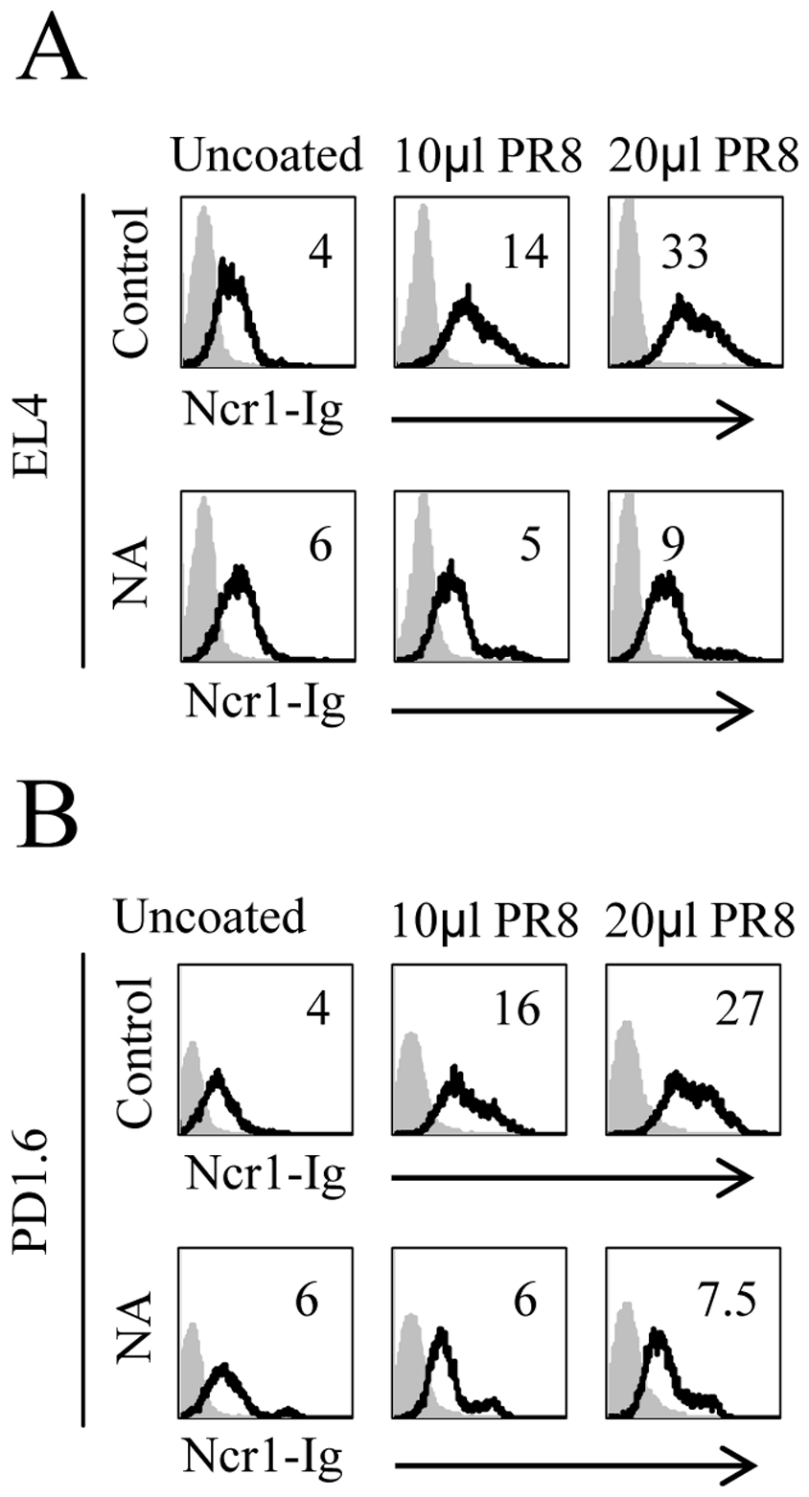
The increased binding of Ncr1-Ig to PR8-coated EL4 and PD1.6 cells is NA-sensitive. The figure shows FACS staining of uncoated (left), PR8 coated 10 µl, 1000 HU (center) and 20 µl, 1000 HU (right) EL4 (**A**) and PD1.6 (**B**). The various cells, untreated, (upper histograms), or NA treated (lower histograms) were stained with Ncr1-Ig (black line), and with a control fusion protein NKp46-D1-Ig (gray filled histograms) Fluorescent intensity (MFI) is presented as the ratio staining/background and is indicated on the upper right side of each histogram. The figure is representative of three independent experiments.

In light of the differences observed in the predicted glycosylation patterns of NKp46 and Ncr1 (Fig. 1), we initially investigated whether the Ncr1 recognition of influenza virus would also be sialic acid dependent. To test this we used the murine carcinogen induced lymphoma EL4 and murine virus-induced thymic lymphoma PD1.6 cells and incubated them with the PR8 influenza virus strain. In this experimental system, the cells do not get infected by the virus, as the coated cells do not undergo spontaneous apoptosis and supernatant taken from the coated cells does not infect MDCK cells (data not shown). Rather, the virus adheres to the cells and this can be detected by the recognition of the virus-coated cells by anti-HA mAb, as soon as one hour after the coating (data not shown). In agreement with previous reports [5], [9], increased binding of Ncr1-Ig was observed to both virus-coated cell lines (Fig. 2). The binding of other fusion proteins such as CD16-Ig, KIR2DL1-Ig, KIR2DL2-Ig, NKp30-Ig, NKG2D-Ig and LIR1-Ig was not increased (data not shown and [6], [9]). Because the increased binding of Ncr1-Ig was reduced following the treatment of the fusion proteins with Neuraminidase (NA) (Fig. 2), we concluded that the Ncr1 recognition of infected cells is sialic acid dependent.

**Figure 3:**
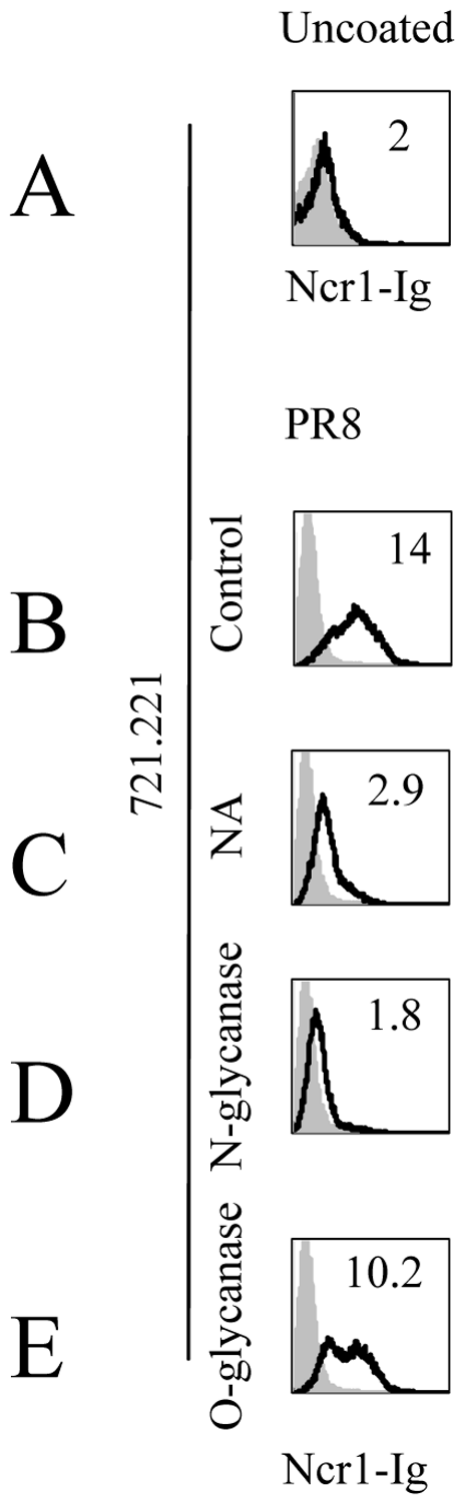
N-glycosylation is required for Ncr1-Ig binding to PR8-coated cells. The figure shows FACS staining of uncoated (**A**) and PR8 coated (20 µl,1000 HU, B-E) 721.221 cells. Staining was performed with Ncr1-Ig (black line), and a control fusion protein NKp46-D1-Ig (gray filled histograms). Coated cells were stained with Ncr1-Ig that underwent various treatments; untreated (**B**), treated with NA (**C**), treated with Protein Glycosidase F (PNGase F) (**D**), treated with a cocktail of O-deglycosylating enzymes (see materials and methods) (**E**). Fluorescent intensity (MFI) is presented as the ratio staining/background and is indicated on the upper right side of each histogram. The figure is representative of three independent experiments.

We next investigated whether the predicted N-glycosylated residues of Ncr1 are involved in its interaction with influenza virus. To test this, we treated the Ncr1-Ig with specific enzymes that hydrolyze either the N-or O-glycosidic linkages. For N-de-glycosylation we used the Protein N-glycosidase F (PNGase F), and for O-de-glycosylation we used a cocktail of four different enzymes (see materials and methods) that were reported to perform exclusive cleavage of O-linked glycans [18]. The various Ncr1-Ig fusion proteins were incubated with PR8-coated 721.221 cells that were used because the 721.221 cells do not express tumor ligands for Ncr1 (Fig. 3A) and because the adherence of the virus and the consequent Ncr1-Ig recognition is efficient in this cell line. In agreement with the above results (Fig. 2), Ncr1-Ig binding was observed to 721.221 cells coated with influenza virus (Fig. 3B) and the increased binding was significantly reduced when the cells were treated with NA (Fig. 3C). Importantly, treatment with N-glycanase (PNGAse F) abolished the Ncr1-Ig binding (Fig. 3D), whereas O-glycanase treatment had only a minimal effect (Fig. 3E). These results indicate that similarly to NKp46, Ncr1 interacts with the influenza virus in a sialic acid dependent manner, but contrary to the NKp46 recognition, the Ncr1 binding of HA is N-glycosylation dependent.

### The Three Glycosylated Residues of Ncr1 are Not Essential for its Influenza Recognition

**Figure 4:**
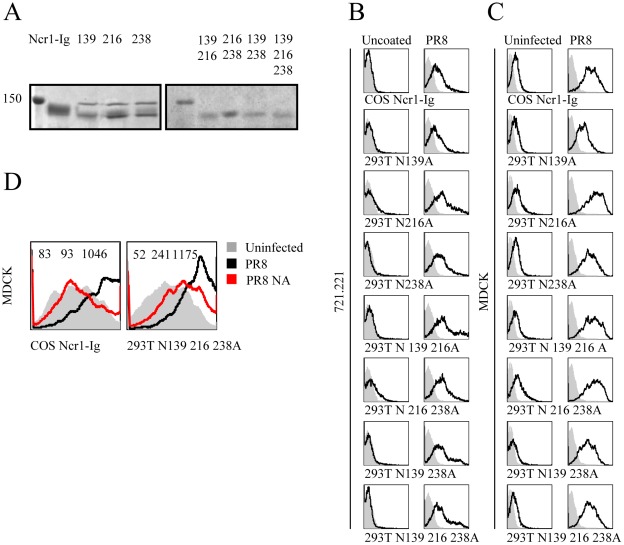
The 3 N-glycosylated residues of Ncr1 are not essential for its interaction with influenza virus. (**A**), SDS-PAGE analysis of the various fusion proteins. First gel (left) featuring from left to right: Ladder, WT Ncr1-Ig fusion protein and Ncr1-Ig with single point mutations, as indicated. Second gel (right) featuring from left to right: Ladder, Ncr1-Ig with double and triple point mutations, as indicated (**B**), FACS staining of uncoated (left lane) and PR8 coated (20 µl, 1000 HU, right lane) 721.221 cells. Staining was performed with a control fusion protein NKp46-D1-Ig (gray filled histograms) with WT Ncr1-Ig generated in COS-7 cells (upper lane), and with Ncr1-Ig generated in HEK293T cells and mutated, as indicated (lower lanes). The figure is representative of three independent stainings. (**C**), FACS staining of uninfected (left lane) and PR8 infected (1000 HU, right lane) MDCK cells. Staining was performed with a control fusion protein NKp46-D1-Ig (gray filled histograms), with WT Ncr1-Ig generated in COS-7 cells (upper lane), and with Ncr1-Ig generated in HEK293T cells and mutated, as indicated (lower lanes). The figure is representative of three independent staining. (**D**) FACS staining of uninfected (filled grey histogram) PR8 infected (black line) or following NA treatment of the fusion proteins (red line) MDCK cells with WT Ncr1-Ig (left) and triple mutated Ncr1-Ig (right). Fluorescent intensity (MFI) of each staining is presented at the top of each histogram. The figure is representative of two independent experiments.

Our next goal was to determine the identity of the specific N-linked glycan residues that are important for HA recognition. For this end we introduced three point mutations in the three Asn residues located in positions 139, 216 and 238 of Ncr1, converting them into Ala. In addition we have generated double (139 216, 216 238, 139 238) and triple mutations (139, 216, 238). We then cloned the mutated Ncr1 genes in frame with human IgG1 and stably transfected them into HEK293T cells. The various fusion proteins were purified on a protein-G column and as can be seen in figure 4A, the purity of all fusion proteins was very high. Furthermore, all the mutated proteins had different electrophoretic mobility, as compared to the wild type Ncr1 protein, suggesting that the predicted residues are indeed glycosylated and that mutating these residues had impaired their glycosylation patterns (Fig. 4A). Similar differences were observed when Ncr1-Ig was produced in COS-7 cells (data not shown), suggesting that the glycosylation pattern of Ncr1 is not substantially different when produced in 293T or in COS-7 cells. Interestingly, the single Ncr1 glycosylation mutants presented a double protein band in the SDS-PAGE gels. This might be because sometimes the abolishment of one glycosylation site can lead to glycosylation changes in other sugar-carrying residues. In contrast, a single protein band was observed in the double and the triple mutated Ncr1 proteins (Fig. 4A), further suggesting that indeed the single mutations led to the aberrant glycosilation of the other residues.

Next, we tested the binding of the mutated and the wild type Ncr1 fusion proteins to 721.221 cells coated with PR8 influenza virus and to MDCK cells infected with PR8 influenza virus (Fig. 4B and Fig. 4C, respectively). Variations were observed in the binding of the various fusion proteins to the infected and to the coated cells (data not shown) and initially it seemed to us that the mutation in each of the predicted N-glycosylated residues of Ncr1 affected its binding. Interestingly however, consistent binding of the double and triple mutated Ncr1 was observed to the coated (Fig. 4B) and to the infected (Fig. 4C) cells, suggesting that none of the N-glycosylated residues of Ncr1 play an essential role in its binding to influenza. Notably, treatment of the wild type Ncr1 fusion protein with NA completely abolished its binding to infected MDCK cells and the NA treatment of the triple mutated Ncr1 fusion protein also reduced its binding significantly but not entirely (Fig. 4D). This suggests that other, yet unidentified glycosylated residues of Ncr1are involved in its influenza virus recognition.

### Binding of Ncr1-Ig to Tumor Cells is Primarily Glycosylation Independent

**Figure 5:**
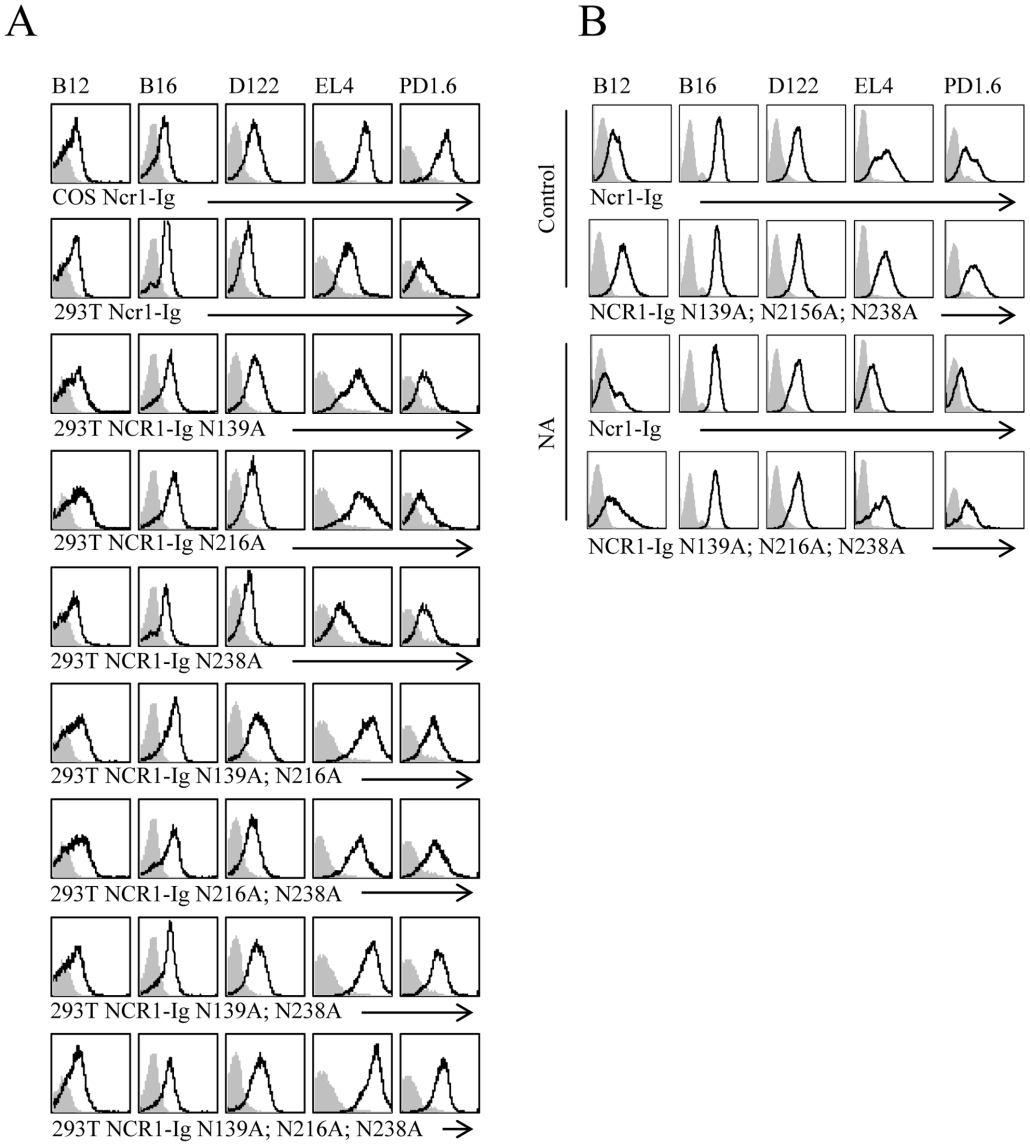
N-glycosylation plays little role in tumor cell recognition. (**A**) FACS staining of various tumor cell lines (indicated above the histograms) with control fusion protein NKp46-D1-Ig (gray filled histograms) and with Ncr1-Ig (black line) generated in COS-7 cells (upper lane), generated in HEK293T cells (second lane up) and generated in HEK293T cells and mutated at the indicated positions (lower lanes). (**B**) FACS staining of various tumor cell lines (indicated above the histograms) with control fusion protein NKp46-D1-Ig (gray filled histograms) with WT (upper and third rows) and triple mutated (second and bottom rows) Ncr-Ig fusion proteins (black line) untreated (two upper rows) or treated with NA (two lower rows).

We have previously shown that glycosylation of NKp46 is important for its binding to some human tumor cell lines [5]. We therefore next tested whether N glycosylation is important for the binding of Ncr1 to mouse tumor cells. The binding of the wild type Ncr1 fusion proteins produced either in COS-7 cells, or in HEK293T to the various cell lines was heterogeneous, probably reflecting the different levels of the unknown Ncr1 tumor ligand(s) on the various cell lines (Fig. 5A). Importantly, little or no change was observed in the binding of the single mutated fusion proteins to the various cells lines. Specifically, while the binding of the single mutated Ncr1 fusion proteins to B16 and D122 did not change, decreased binding of the single mutated Ncr1 fusion proteins to PD1.6 cells was observed (Fig. 5A). Interestingly, however, the binding of the double and triple mutated Ncr1 fusion proteins to all the cell lines was unchanged or even increased, suggesting that the 3 N-glycosylated residues of Ncr1 play little role in the recognition of the tested tumor cell lines. Indeed, treatment of the WT Ncr1 and the triple mutated Ncr1 fusion proteins with NA also had minimal effect on their binding (Fig. 5B) and in all cases the NA treatment did not completely abolish the Ncr1 binding (Fig. 5B).

### Ncr1 is Essential for Influenza Virus Killing *in-vitro* and *in-vivo*

**Figure 6:**
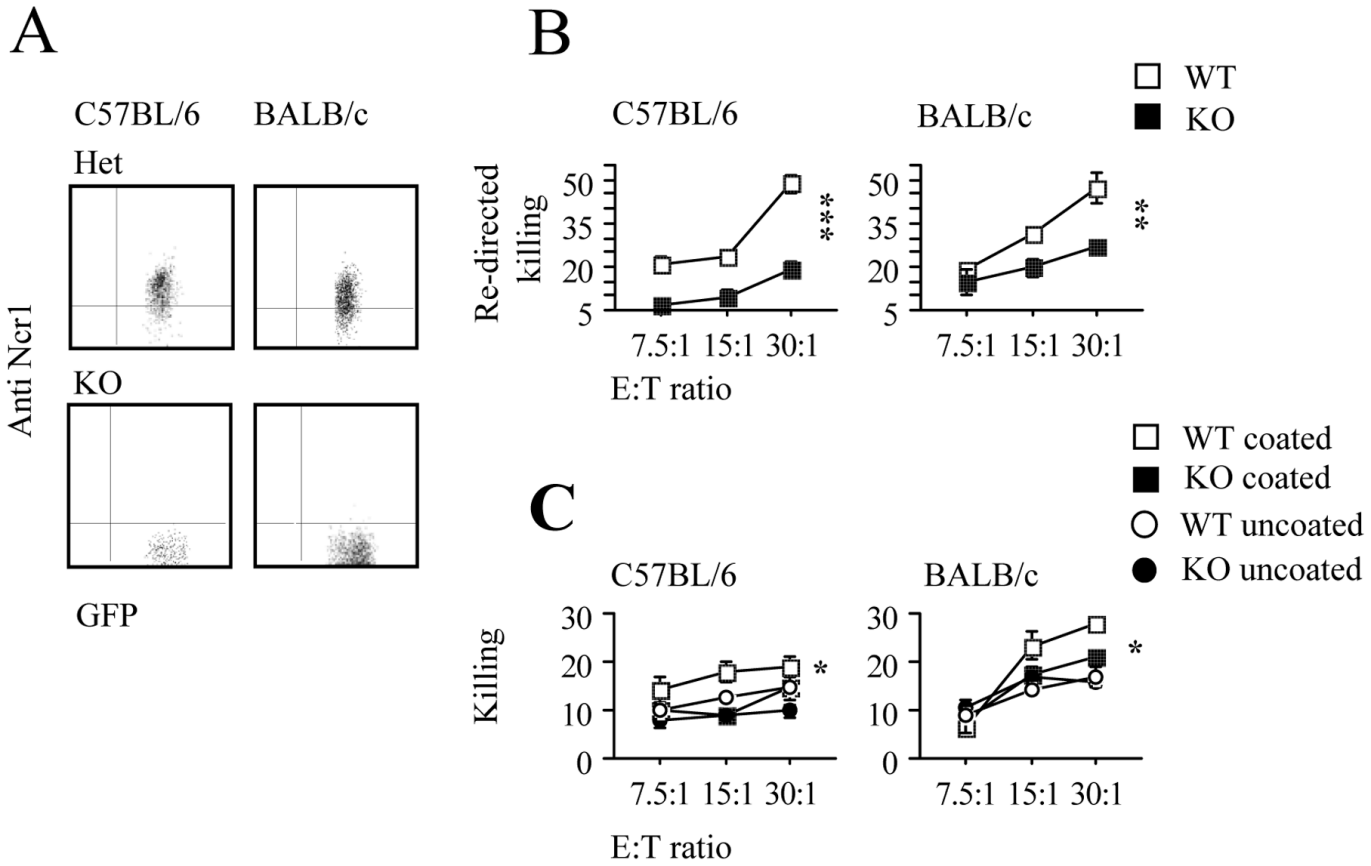
Ncr1 mediated killing. (**A**) FACS staining of PBLs with Ncr1 mAb. The figure shows the intrinsic GFP expression of the NK cells and is representative of two independent staining. (**B**) Line chart depicting 35S methionine Re-directed killing assay. Labeled p815 cells were pre-incubated with anti-Ncr1 mAb and then incubated with PBLs harvested 18 hours following 200 µg poly(I):poly(C) in 200 µl PBS administered by i.p. injection to WT and KO mice of the C57BL/6 and BALB/c strains. Values are shown as mean ±SEM. The figure is representative of two independent experiments. ***; P<0.0005, **; P<0.005 (**C**) line chart depicting 35S methionine killing assay. PBLs were harvested 18 hours following 200 µg poly(I):poly(C) in 200 µl PBS administered by i.p. injection to WT and KO mice of the C57BL/6 and BALB/c strains and incubated with labeled EL4 cells. At least 8 mice were used in each group; a representative of three independent experiments is shown. Values are shown as mean ±SEM P<0.05.

We have previously shown by using two mouse strains (C57BL/6 and 129/Sv) that in the absence of Ncr1 influenza virus infection is lethal [4]. To test whether Ncr1 would be important for influenza virus recognition in an environment dominated by Th2 cytokines, we crossed the C57BL/6 *Ncr1gfp/gfp* (KO) mice that we have generated [4] with BALB/c mice for more than 10 generations. Since we knocked out *Ncr1* by replacing it with *GFP*, all the NK cells in the Ncr1 KO and in the heterozygous (Het) mice are GFP positive. Indeed, as can be seen in figure 6A, which shows staining of the KO and Het mice of the C57BL/6 and BALB/c strains, all NK cells in the Het mice express GFP and are Ncr1 positive, while the KO NK cells are GFP positive, Ncr1 negative. We further verified that Ncr1 is not functional in the C57BL/6 and BALB/c strains by using a redirected killing assay (see Materials and Methods section). P815 cells were pre-incubated with anti-Ncr1 mAb and then incubated with PBLs taken from the different mice. Figure 6B shows that the activation of Ncr1 by the specific antibody resulted in the re-directed killing of P815 cells by WT PBLs derived from C57BL/6 and BALB/c mice, while the killing of P815 cells was much reduced when PBLs were taken from the KO mice of either background. The little killing of P815 cells observed with the KO PBLs is probably mediated by NK killer receptors other than Ncr1.

To test whether NK cells of WT and KO mice of the C57BL/6 or BALB/c strains would efficiently kill murine cell lines coated with influenza virus *in-vitro*, we performed direct 35S-methionine cytotoxicity assays. Untreated and PR8 coated and 35S-methionine labeled EL4 cells were incubated with PBLs derived from WT and KO mice of the C57BL/6 and BALB/c backgrounds. As mentioned above, the EL4 cells were not infected with influenza as they did not undergo spontaneous apoptosis following incubation with the virus and supernatants taken from the EL4 cells coated with influenza were unable to infect MDCK cells (data not shown). As can be seen in figure 6C, NK cells of both strains killed the virus-coated cells to a significantly higher extent (coated, uncoated; P<0.05 in both strains), and in both strains the killing was partially Ncr1 mediated, as NK cells derived from the WT mice killed the coated target cells significantly better than NK cells derived from the KO mice (WT, KO; P<0.05 in both strains).

**Figure 7:**
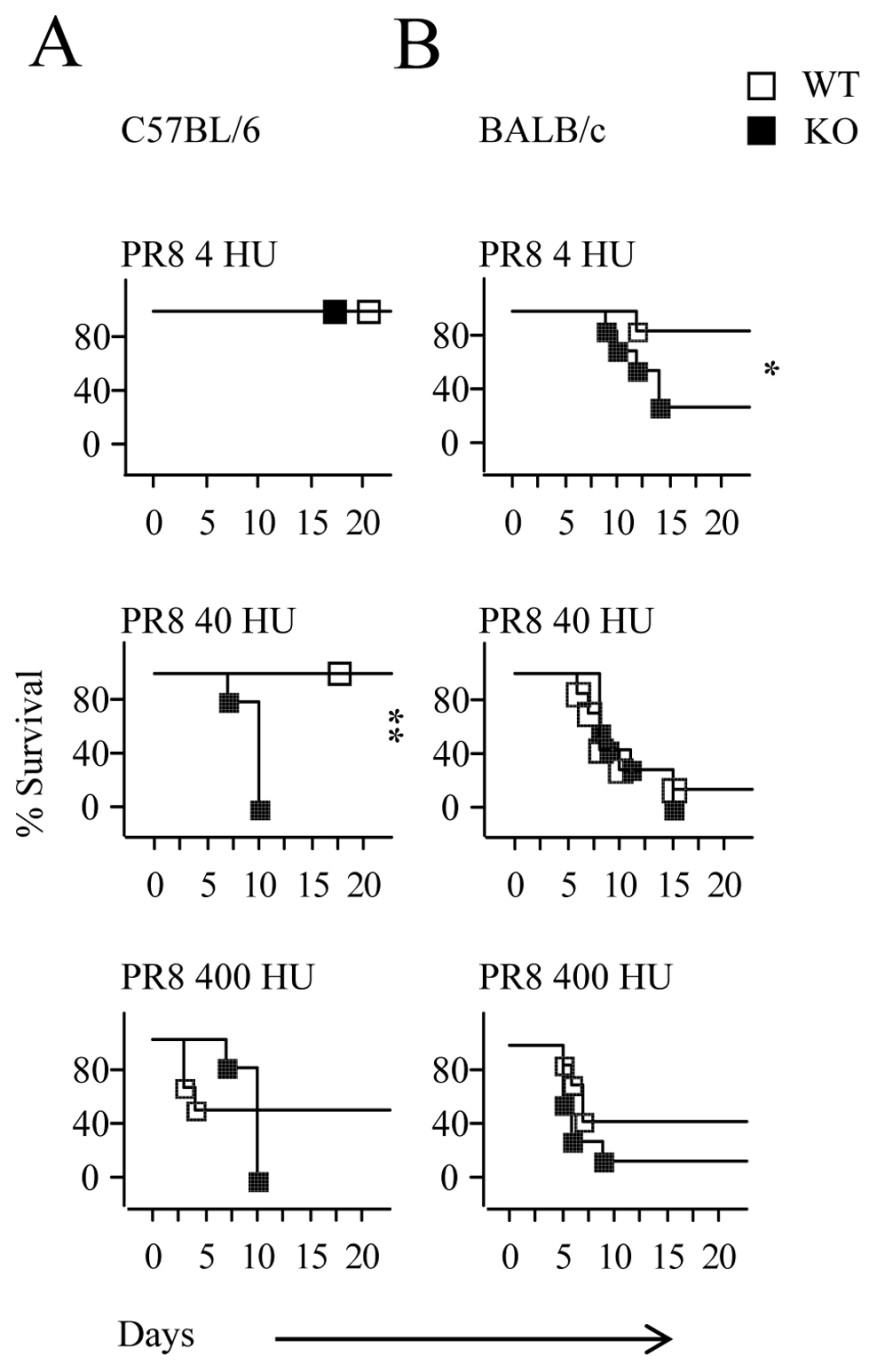
Ncr1 is critical for *in-vivo* influenza eradication. C57BL/6 (**A**) and BALB/c (**B**) WT and KO mice were infected with two doses of PR8 influenza, as indicated. Survival was assessed using the Kaplan Meier model and the Tarone-Ware test. Termination point of the experiment was set to 20 days and mice that survived the infection were then considered healthy. The figure represents one of two independent experiments; at least 7 mice were used in each group. *P<0.05, **P<0.005.

We have shown that the killing of PR8 coated cells is Ncr1-dependent and that influenza virus infection is lethal in C57BL/6 or 129/Sv mice [4]. To test whether Ncr1 is also important for influenza virus killing *in-vivo*, in the Th2 oriented BALB/c background [21], we intranasaly infected KO and WT C57BL/6 and BALB/c mice with three doses of PR8 influenza virus, and monitored mortality. In the C57BL/6 mice, at the low dose of influenza virus infection (4HU) no mortality incidence was observed (100% survival). However, in accordance with our previous report [4], in the higher dose of influenza virus infection (40 HU), differences between the WT and KO mice were clearly evident and while no mortality was observed in the WT mice, 100% of the KO mice succumbed to the infection and died (P<0.005) (Fig. 7A).

Notably, influenza virus infection in BALB/c mice was different than in the C57BL/6 mice. The BALB/c mice seemed to be more sensitive to the infection as even at the low dose of influenza virus (4 HU), nearly all the KO mice succumbed to the infection, as evident by weight loss (data not shown) and mortality (Fig. 7B) and even a small percent of the WT mice died. Still, however, in this dose, a significant difference was evident between the WT and KO mice in resistance to influenza virus infection (P<0.05). Interestingly, at the higher dose of influenza virus infection (40HU), all BALB/c mice were infected, and mortality was equally high in the WT and KO mice (Fig. 7B).

To better compare the Ncr1-dependency of the C57BL/6 and the BALB/c mice we used the LD50 400 HU to infect the mice (Fig. 7A and Fig. 7B). In both strains almost all the KO mice had died, although the differences were not statistically significant. Interestingly, in the BALB/c strain, mortality of the WT mice was higher when less HU were administered, as mortality was near 100% at 40 HU, but reached only 60% at 400 HU (please compare infection of BALB/c mice with 40HU versus 400 HU in figure 7B). This difference (that was not statistically significant) might be due to the generation of virus complexes at high HU which reduces the efficiency of the infection.

## Discussion

Influenza virus infection poses one of the major threats to modern society [22]. Several major pandemics occurred in the last two centuries; the 1918 Spanish pandemic (A/H1N1), the 1956 Asian flu (H2N2), the 1968 Hong Kong flu (H3N2) and the 2009 Swine flu (H1N1-2009) [23]. Therefore, studying influenza virus and its interactions with the immune system is of extreme importance. With this regard, it is also imperative that we understand whether the interaction of the mouse immune system with influenza virus is similar to the humans’ and whether the experiments performed in mice are relevant for human infection.

NK cells are proficient in the eradication of a variety of virus-infected and tumor cells [1], [2], [3]. Their ability to kill hazardous cells stems, in many cases, from the activation of their killer receptors [1], [2], [3]. We have shown that the interaction between NKp46/Ncr1 and viral HA is essential for the control of influenza virus infection *in-vitro* and *in-vivo,* and have demonstrated that sialic acid residues are important for the binding of NKp46 [4], [5], [9].

Sialic acids are usually added to N- or O-linked glycans by sialyltransferases, in either an α2–3linkage or an α2–6 linkage. O-linked glycans that are acquired in the Golgi, on serine or threonine residues, are less abundant than N-linked glycosylations. O-linked glycans have a role in the binding of glycoproteins and in the masking of the protein backbone [24].

N-linked glycans are attached to asparagine nitrogen groups and are composed of N-acetyl galactosamine, galactose, neuraminic acid, N-acetylglucosamine, fructose, mannose, fucose, or other monosaccharides assembled in the cytoplasm and endoplasmic reticulum [24]. It was recently reported that N-glycans are crucial in influenza virus infection and entry into the host cells, irrespective of O linked glycans, or sialic acids contents [25].

We have shown that the binding of NKp46 to the influenza virus HA is sialic acid and O-glycosylation dependent [5], [6], [9]. In this study, we demonstrate that the Ncr1 recognition of influenza virus is also sialic acid mediated and that it is N-glycosylation dependent. Surprisingly, none of the predicted N-glycosylated residues of Ncr1 were shown to be essential for this binding.

We demonstrated that treatment of the triple mutated Ncr1 protein with NA reduced its binding to the infected cells and that N-glycanase treatment completely abolished the binding of Ncr1 to the virus-infected/coated cells. These findings suggest that other, yet unidentified N-glycosilation residues of Ncr1 are important for its binding to the influenza virus. The identity of these residues is currently unknown. An initial bioinformatics search revealed that 3 potential N glycosylated residues are located at the membrane distal domain of Ncr1. Future research (which is beyond the scope of this manuscript) will determine whether these residues are indeed glycosylated and whether they are essential for the Ncr1 recognition of infected cells.

We suggest that the NA treatment did not completely abolish the triple Ncr1 mutant binding to infected cells probably because the removal of the sugars allowed a tighter binding of the fusion proteins to the negatively charged membrane. This is also consistent with the unchanged or increased binding of the double and triple mutated fusion proteins that is observed to the tumor cells. Alternatively, it is possible that other elements, which are exposed following the removal of the sugars in the mutated Ncr1-Ig protein are responsible for the better binding of the double and triple mutated Ncr1 proteins to PR8 coated and infected cells and to some tumor cells. Supporting the later option are the observations that although almost all proteins carry sialic acids, the recognition of influenza by Ncr1 and NKp46 seems to have a certain unique specificity, as other glycosylated cell receptors such as KIR2DL1, KIR2DL2, NKp30, CD16, NKG2D and LIR1([6], [9] and data not shown) do not demonstrate increased binding to the infected cells.

HA is used by the virus to bind host cell proteins [23]. In some of the experimental systems we used here the cells were not infected by the virus but rather the virus only adhered to the cells. Nevertheless, NK cells were able to kill these virus-coated cells. This suggests that NK cells are capable of killing cells as soon as they are contacted by the influenza virus, even before they are actually infected. Such early killing would be very effective in preventing infection.

Human viruses recognize N-acetyl sialic acids linked to galactose with an α2,6 linkage in the upper respiratory tract, such as 6′SLN, and 6′SLN-LN, whereas avian viruses recognize receptors containing N-acetyl sialic acids linked to galactose by an α2,3 linkage such as 3′SLN-LN and 3′SLN-LN-LN [26]. The sialic acid binding domain of the HA protein is therefore essential for the infectivity and virulence of the virus [27]. Indeed, although the HA protein undergoes extensive variations, the sialic acid binding capacity of the HA is conserved [27], [28]. This property, as we show here and previously [4], [5], [9], is elegantly used by the NKp46/Ncr1 to kill the infected cells via the sialic acid-dependent mechanism, thus enabling NK cells to kill many different influenza virus strains.

Viral HA is not the only ligand of the NKp46/Ncr1, as we have previously shown [8], [10], [11], [13]. Similar pleura of ligands is observed with regard to other NK cell receptors such as NKG2D, which recognizes eight different ligands (MICA, MICB and ULBP 1–6 [29]), and NKp30 which recognizes the pp65 protein of HCMV [30], BAT3 [31], B7-H6 [32], and another unknown ligand expressed by DCs [33]. This model of a single receptor that recognizes several ligands enables the innate immune system to control a wide range of pathogens and diseases.

In our previous work, we have demonstrated the importance of Ncr1 in the control of influenza virus *in-vivo*, by comparing WT and Ncr1 KO lines in two inbred mouse strains, the 129/Sv [4] (in which signaling via the Ly49-activating receptor is impaired [34]), and the immune-competent C57BL/6 strain. Here we tested whether Ncr1 would be important for influenza virus eradication in a BALB/c mouse that has a Th2 oriented immune environment [21]. Interestingly, the function of Ncr1 in the BALB/c background seems to be less important with regard to influenza virus infection and these mice were indeed more sensitive to the infection. Similar differences were observed also with regard to other receptors and other viruses such as NKG2D and MCMV [35], [36]. The difference in sensitivity of the two mice strains to influenza virus infection is probably due to the innate resistance of the C57BL/6 strain to intracellular pathogens, associated with increased activity of Th1 cells, the induction of Th1 oriented responses and the activation of cell mediated immunity [37], [38]. Conversely, BALB/c mice are more susceptible to intracellular pathogens, as their primary response is an anti-inflammatory Th2 type, which is aimed against extracellular pathogens and involves humoral immunity [38], [39].

Finally it was recently shown that the impaired cell surface expression of Ncr1 in a mouse named Noé caused a receptor-independent, hyper reactive phenotype [40]. Some of these findings were also reproduced in a mouse in which the iCre gene was inserted in the 3′ UTR of Ncr1, resulting in reduced mRNA levels of *Ncr1* and impaired surface expression [40]. In contrast, the *Ncr1gfp/gfp* KO mice that we have generated do not show this hyper-reactivity [4] and we and others have shown that only their *Ncr1*-mediated activities are impaired [4], [7], [8], [10], [11], [12], [13].

The various Ncr1 mice are substantially different. Firstly, the *Ncr1gfp/gfp*, Noé and *Ncr1* iCre mice differ in the intracellular levels of Ncr1. The *Ncr1gfp/gfp* has no intracellular Ncr1 expression while the Noé mouse was reported to have intracellular expression of the full length Ncr1 protein [40]. The *Ncr1* iCre mouse was reported to have reduced levels of the *Ncr1* transcript, but no cell surface expression, suggesting that in this mouse the full length *Ncr1* protein is found inside the cell as well. Thus, we propose a possibility in which the cytoplasmatic arrest of the full length *Ncr1* protein determines whether the NK cells will be hyper-reactive (Noé and *Ncr1* iCre) or will have impaired Ncr1-mediated activities (*Ncr1gfp/gfp*).

The other major difference between the *Ncr1gfp/gfp*, the Noé and the *Ncr1* iCre mice is the presence (Noé and *Ncr1* iCre), or the absence (*Ncr1gfp/gfp*) of a full length *Ncr1* gene. We have knocked out *Ncr1* by replacing exons five-seven with *GFP* [4]. In contrast, the complete *Ncr1* gene is present in both the Noé (as only a single mutation was introduced) and in the *Ncr1* iCre mice (as the iCre gene was inserted in the 3′ UTR of Ncr1 [40]). Therefore, it is possible that the missing part of the *Ncr1* gene in the *Ncr1gfp/gfp* mice contains certain regulatory elements that are involved in the regulation of the NK function under certain conditions and this is the cause for the differences observed between the various mice. Regardless of the reasons accounting for the different phenotypes observed in the various Ncr1 KO mice, it is clear that the *Ncr1gfp/gfp* are best suitable for studying the outcome of specific functions of Ncr1.
